# Infanticide as a male reproductive strategy has a nutritive risk effect in brown bears

**DOI:** 10.1098/rsbl.2013.0624

**Published:** 2013-10-23

**Authors:** S. M. J. G. Steyaert, C. Reusch, S. Brunberg, J. E. Swenson, K. Hackländer, A. Zedrosser

**Affiliations:** 1 Institute of Wildlife Biology and Game Management, University of Natural Resources and Life Sciences, Vienna 1180, Austria; 2 Department of Ecology and Natural Resource Management, Norwegian University of Life Sciences, 1432 Ås, Norway; 3 Norwegian Institute for Nature Research, 7485 Trondheim, Norway; 4 Department of Environmental and Health Studies, Telemark University College, 3800 Bø, Norway

**Keywords:** brown bear, risk effects, segregation, sexually selected infanticide

## Abstract

Behavioural strategies to reduce predation risk can incur costs, which are often referred to as risk effects. A common strategy to avoid predation is spatio-temporal avoidance of predators, in which prey typically trade optimal resources for safety. Analogous with predator–prey theory, risk effects should also arise in species with sexually selected infanticide (SSI), in which females with dependent offspring avoid infanticidal males. SSI can be common in brown bear (*Ursus arctos*) populations and explains spatio-temporal segregation among reproductive classes. Here, we show that in a population with SSI, females with cubs-of-the-year had lower quality diets than conspecifics during the SSI high-risk period, the mating season. After the mating season, their diets were of similar quality to diets of their conspecifics. Our results suggest a nutritive risk effect of SSI, in which females with cubs-of-the-year alter their resource selection and trade optimal resources for offspring safety. Such risk effects can add to female costs of reproduction and may be widespread among species with SSI.

## 1. Introduction

Infanticide is common among mammals and can be a male reproductive strategy (sexually selected infanticide, SSI) provided that three requirements are fulfilled, that is (i) males only kill offspring that they have not fathered, (ii) victimized mothers enter oestrus shortly after offspring loss and (iii) the perpetrating male has a high probability of fathering the victimized mother's next offspring [1]. SSI has been documented in a variety of species and guilds, and is especially common in polygamous, size-dimorphic species with extended maternal care and lactational anoestrus [2,3]. In seasonal breeders, SSI has the highest potential benefit when committed during the mating season and only if victimized females rapidly re-enter oestrus. Because SSI has an obvious fitness cost for females, various counter strategies for SSI have evolved, including multi-male mating, grouping and spatio-temporal avoidance [4]. Evidence for spatio-temporal avoidance to reduce SSI risk is rare [4], but suggestive evidence has been found in, for example, lions (*Panthera leo*) [5] and brown bears (*Ursus arctos*) [6,7].

Predator–prey theory postulates that antipredator adaptations can be costly (e.g. risk effects) [8]. Many species reduce predation risk by trading off optimal energetic resources for safety by spatio-temporal avoidance of the risk source [8,9]. Because such trade-offs are directly related to fitness, prey can incur a significant fitness cost due to predation risk [10]. If predation risk is predictable, animals can respond rapidly to changing predation risk regimes [9] and select their resources in such a way to bridge risk periods, irrespective of the instantaneous risk (i.e. the risk spreading theorem) [11]. After a predictable ‘high-risk’ period, animals can compensate for the energetic cost of reduced feeding by feeding more intensively or selecting higher quality foods [9]. Analogous with predator–prey theory, a trade-off between selecting optimal foods or safety should exist in relation to SSI [6].

Here, we evaluate if spatio-temporal avoidance of SSI may have a nutritive risk effect, by using the brown bear as a model species. Infanticide can be common in brown bear populations and is almost exclusively committed by males during the mating season [2]. Strong support for SSI exists from some brown bear populations [2,12], and females use various strategies to reduce the risk of infanticide, including spatio-temporal avoidance of conspecifics [6,7]. We compared diet quality of females with cubs-of-the-year (‘females/cubs’, greater than or equal to 5 years, accompanied by cubs-of-the-year), lone females (greater than or equal to 5 years) and adult males (greater than or equal to 5 years) between the mating and the post-mating season (i.e. high versus no SSI risk) in a population where SSI is common [2], and predicted that females/cubs have (i) lower quality diets than conspecifics during the mating season as a result of spatio-temporal SSI avoidance strategies, but (ii) not during the post-mating season, when diet quality of females/cubs should be at least as good as the diet quality of conspecifics.

## 2. Material and methods

### (a) Study area

Our study was conducted in a boreal forest in Dalarna and Gavleborg counties, Sweden. The study area encompassed approximately 13000 km^2^, and the bear density was approximately 30 individuals per 1000 km^2^ [6].

### (b) Diet quality data

The chemical composition of forage and faeces derived from that forage is strongly correlated, and measures derived from faeces can serve as proxies for diet quality [13]. The digestive efficiency of brown bears is similar to those of obligate carnivores, and bears are not adapted to efficiently digest coarse forage [14]. Therefore, we used % dry faecal matter of faecal crude fibre content (FCF) and faecal protein content (FP) as proxies of diet quality in bears. High-FCF content indicates low diet quality, whereas high-FP content indicates high quality [14].

We collected faeces from Global Positioning System (GPS; Vectronic Aerospace GmbH) collared bears of known sex and age (i.e. known year of birth or cementum age analysis) during the mating season (1 May–15 July) and post-mating season (1 August–30 September) of 2010. We sampled faeces from each bear at location cluster sites (greater than or equal to three consecutive 30 min GPS positions within a 30 m radius), and used near-infrared spectroscopy to measure FCF and FP content. To control for possible methodological effects on FCF and FP content, we recorded the maximum field exposure time (h), canopy cover (% cover, measured with a densiometer) and oven drying time (h) for each sample. Refer to Steyaert *et al.* [15] for methodological details.

### (c) Statistical analysis

We used linear mixed-effect regression models to test our predictions with FCF and FP content as response variables. We included ‘bear ID’ as a random component and considered ‘reproductive state’, ‘season’, ‘reproductive state × season’, ‘canopy cover’, ‘field exposure time’, ‘canopy cover × field exposure time’ and ‘drying time’ for inclusion in models. We considered ‘age’, ‘age × reproductive state’ and ‘age × season’ for inclusion in our models to account for age-related behavioural differences. We used second-order, bias-corrected Akaike's information criteria differences (*Δ*AIC_c_) and weights (AIC_cw_) for model selection [16]. We averaged models with *Δ*AICc values less than 2 [16]. We evaluated FCF and FP by running all possible combinations of model terms (*n* = 1024), with the expectation that ‘reproductive state’, ‘season’ and ‘reproductive state × season’ would be included in the top-ranked models. We used R v. 2.12.0 [17] for statistical analysis.

## 3. Results

We obtained FCF and FP measurements of 491 samples from 12 lone females (*N*_mating season_, *N*_m_ = 139, *N*_postmating season_, *N*_m_ = 95), 11 adult males (*N*_m_ = 71, *N*_pm_ = 64) and nine females/cubs (*N*_m_ = 102, *N*_pm_ = 20). Average time between two samples collected from the same bear was 7.5 days. We classified two females/cubs that lost their entire litter due to infanticide during the mating season as ‘lone females’ after the loss.

**Figure 1.**
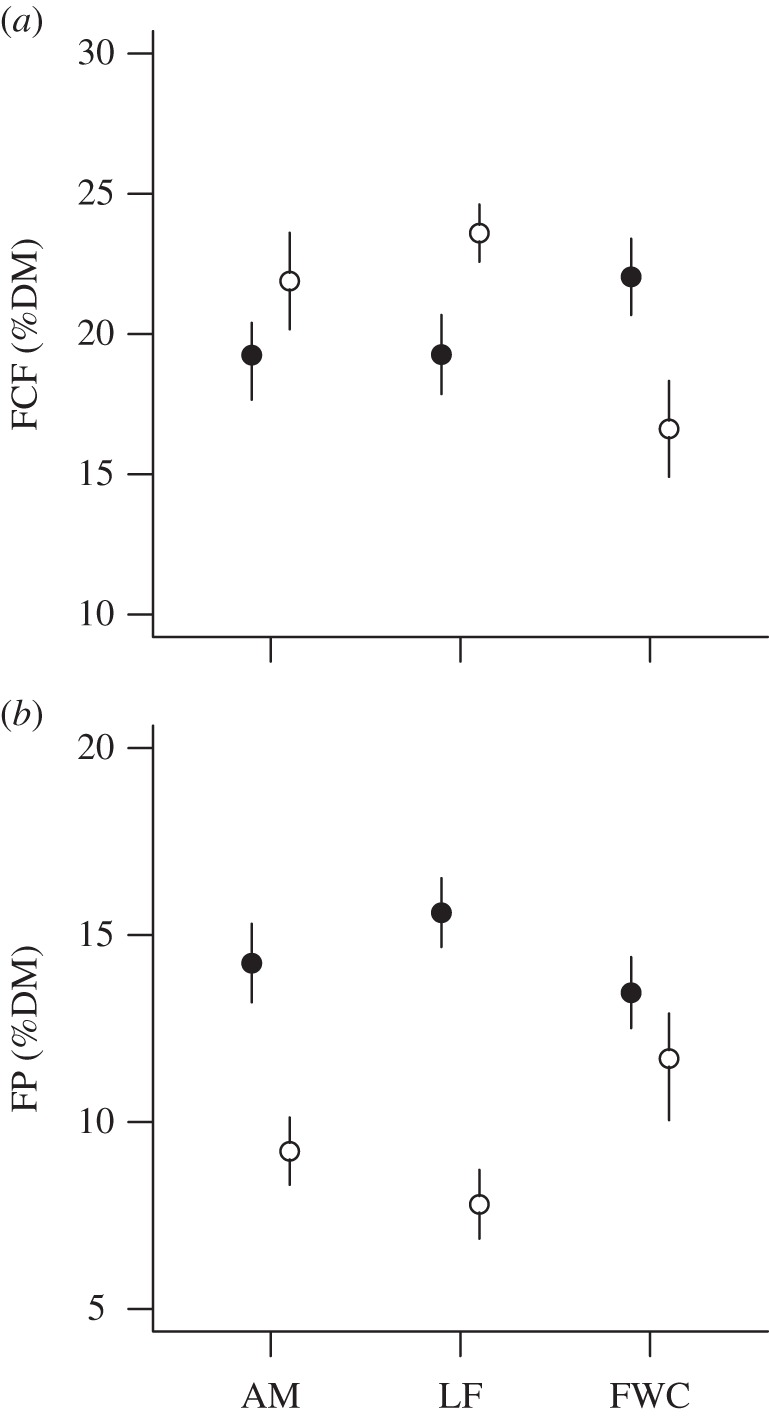
(*a*) Predicted FCF and (*b*) FP content in brown bear faeces (adult males (AM), lone females (LF) and females with cubs-of-the-year (FWC)), collected in south–central Sweden in 2010 during the mating (filled circles) and post-mating (open circles) season. Whiskers indicate one standard error. High FCF content indicates low diet quality; high FP content indicates high quality.

All models with *Δ*AICc values less than 2 to predict FCF (six models) and FP (five models) contained ‘reproductive state’, ‘season’ and ‘season × reproductive state’, and accounted for 77% and 74% of the AIC_cw_ of all possible model combinations, respectively. These models always included the methodological controls ‘canopy cover’, ‘field exposure time’ and ‘drying time’, and sporadically included ‘age’, ‘age × season’, ‘age × reproductive state’ and ‘field exposure × drying time’ (see electronic supplementary material). After model averaging, the FCF content during the mating season was higher for females/cubs than for lone females (*β* = 2.77, *σ* = 1.36, *p* = 0.042), but not different from adult males (*β* = 2.79, *σ* = 1.6, *p* = 0.082). FCF content during the post-mating season was lower for females/cubs compared with lone females (*β* = −6.98, *σ* = 1.71, *p* < 0.001) and adult males (*β* = −5.265, *σ* = 1.29, *p* = 0.004; figure 1*a* and electronic supplementary material). FP content during the mating season was lower for females/cubs than for lone females (*β* = −2.14, *σ* = 0.95, *p* = 0.024), but not for adult males (*β* = −1.003, *σ* = 0.99, *p* = 0.31). FP content during the post-mating season was higher for females/cubs than lone females (*β* = 3.92, *σ* = 1.02, *p* = 0.001), but not compared with adult males (*β* = 2.5, *σ* = 1.28, *p* = 0.052; figure 1*b* and electronic supplementary material). No heteroskedasticity was apparent in the models.

## 4. Discussion

Diet quality of bears in our study system varied across seasons and reproductive classes. As predicted, females/cubs generally had lower quality diets than conspecifics during the high-risk period; whereas their diet quality was at least as good as that of conspecifics when the risk for SSI was nearly absent. Our results suggest that spatio-temporally avoiding SSI has a nutritive cost. Females/cubs had diets of better quality than lone females during the post-mating season, suggesting that they compensated for the energetic costs by selecting higher quality foods during the post-mating season [9]. Diet quality differences between adult males and lone females were less pronounced. Because competition for mates is stronger among males than females, we suggest that males trade feeding for mate acquisition during the mating season [6]. The generally higher FP values during the mating season probably reflected the seasonal availability of vulnerable moose (*Alces alces*) calves. Predation risk effects have been widely studied, and evidence for the food-or-safety trade-off exists for many species [8–10], however, not in relation to the functional significance of infanticide.

Alternative explanations for the observed differences in diet quality among the seasons and reproductive classes include sexual dimorphism, cub mobility and physiological aspects of maternal care. However, we did not find differences in diet quality between lone females and adult males during the two seasons, suggesting that sexual dimorphism, related nutritive needs and competitive abilities probably did not cause segregation in our study system. Cub mobility is unlikely to constrain maternal foraging behaviour. Martin *et al*. [18] showed that females/cubs more often made long-distance movements prior to than during the mating season and suggested that females/cubs adapt to an elusive lifestyle during the mating season to reduce infanticide risk.

Physiological aspects of lactation and maternal care are also unlikely to explain the observed patterns in diet quality across reproductive classes and seasons. Food digestibility is directly related to diet composition in bears, and no differences in food digestibility across sexes, species and taxonomic groups of carnivores are apparent [14]. If physiological aspects of lactation were to affect diet quality in females/cubs, we would not have expected a pronounced seasonal change in their diet quality, because lactation lasts up to 2.5 years and peaks during the cubs' first summer, i.e. after the mating season [19].

In the realm of optimal foraging and predator-prey theory, it is not surprising that a food-or-safety trade-off emerges from intraspecific forms of predation, for instance infanticide. We suggest that a nutritive risk effect can be widespread among species in which SSI is an important source of offspring mortality. SSI can directly affect female reproductive success, and can greatly increase the *per capita* energetic investment in offspring. This energetic investment, in turn, can be constrained by infanticide risk. Our results show that risk effects of SSI can add to the female costs of reproduction. However, the extent of that cost yet remains unknown.

## Data accessibility

These data are deposited in the Dryad repository: doi:10.5061/dryad.h359g [20].
